# An In-Depth Exploration of Knowledge and Beliefs Associated with Soda and Diet Soda Consumption

**DOI:** 10.3390/nu12092841

**Published:** 2020-09-17

**Authors:** Caroline Miller, Kerry Ettridge, Melanie Wakefield, Simone Pettigrew, John Coveney, David Roder, Sarah Durkin, Gary Wittert, Jane Martin, Joanne Dono

**Affiliations:** 1 School of Public Health, The University of Adelaide, Adelaide, SA 5000, Australia; 2 Health Policy Centre, South Australian Health and Medical Research Institute, Adelaide, SA 5000, Australia; Kerry.ettridge@sahmri.com (K.E.); Jo.dono@sahmri.com (J.D.); 3 School of Psychology, The University of Adelaide, Adelaide, SA 5000, Australia; 4 Centre for Behavioural Research in Cancer, Cancer Council Victoria, Melbourne, VIC 3004, Australia; melanie.wakefield@cancervic.org.au (M.W.); sarah.durkin@cancervic.org.au (S.D.); 5 School of Psychological Sciences, The University of Melbourne, Melbourne, VIC 3010, Australia; 6 Food Policy, The George Institute for Global Health, Sydney, NSW 2042, Australia; SPettigrew@georgeinstitute.org.au; 7 College of Nursing and Health Sciences, Flinders University, Adelaide, SA 5042, Australia; john.coveney@flinders.edu.au; 8 Cancer Epidemiology and Population Health, University of South Australia, Adelaide, SA 5000, Australia; david.roder@unisa.edu.au; 9 Discipline of Medicine, University of Adelaide, Adelaide, SA 5000, Australia; gary.wittert@adelaide.edu.au; 10 Centre for Nutrition and GI Diseases, South Australian Health and Medical Research Institute, Adelaide, SA 5000, Australia; 11 Obesity Policy Coalition and Alcohol and Obesity Policy, Cancer Council Victoria, Melbourne, VIC 3004, Australia; jane.martin@cancervic.org.au

**Keywords:** sugar-sweetened beverages, artificially sweetened beverages, population survey, knowledge, beliefs, consumption

## Abstract

The need to reduce sugar-sweetened beverage (SSB) consumption is widely accepted, but whether artificially sweetened beverages (ASBs) are a recommended alternative is a growing policy issue because of emerging evidence of potential health effects associated with excess consumption. This study aimed to establish the extent of the Australian population’s knowledge of the risks associated with consuming SSBs (e.g., soda) and ASBs (e.g., diet soda), which is essential for identifying which facets of knowledge to target with public health interventions. A national computer-assisted telephone survey of 3430 Australian adults was conducted in 2017. The survey included a range of measures to test associations between SSB and ASB knowledge and beliefs, demographic characteristics, and soda and diet soda consumption. Participants had an overall awareness that there were health risks associated with SSB and ASB consumption, but they lacked more detailed knowledge of health effects and nutritional composition of these drinks. These knowledge gaps are concerning given that SSBs and ASBs are consumed in large quantities in Australia. Public health interventions targeting consumers’ limited knowledge and perceptions of health risks associated with excess sugar, calorie intake and artificial sweeteners are essential in reducing the health burden of obesity.

## 1. Introduction

Curbing population consumption of sugar-sweetened beverages (SSBs) is a public health priority [1]. In Australia, like many other countries, SSB consumption is prevalent [1]. Nearly half of the adult population consumes SSBs regularly, with consumption highest among adolescents and young adults (14–24 years) [2,3]. SSBs are problematic because they are often consumed in high volumes, offer no nutritional value and are associated with a range of health effects [4,5,6,7,8,9,10,11]. Sodas, as a subgroup of SSBs that are distinct from juice and energy/sports drinks, are especially problematic because of their market dominance in Australia and globally [1].

Encouraging increased consumption of water, preferably fluoridated tap water, is the recommended alternative [12]. However, there are many substitutes for SSBs, including artificially sweetened beverages (ASBs). No- or low-sugar versions of beverages are heavily promoted by industry as an alternative to SSBs. Adding further complexity, there is now a body of research suggesting that excess consumption of ASBs may also lead to increased health risks, notably weight gain and Type 2 Diabetes [13,14]. While the strength of this evidence is currently limited due to methodological and reporting quality [15], it is an active area of research that has important policy implications [16]. For example, an intervention such as a tax that is applied to SSBs but not ASBs may inadvertently or intentionally nudge consumers towards higher consumption of ASBs rather than water. Adding further confusion is public concern that artificial sweeteners increase the risk of cancer in humans, although, there is no clear evidence supporting this concern [17]. Globally, Australians are among the highest consumers of ASBs, with sales data from 2000 to 2014 indicating that consumption rates are increasing [1]. The extent to which Australians perceive regular ASB consumption as a health risk is currently unknown.

The proposition that people should ‘make healthier choices’ is predicated on the availability of accessible and interpretable information to inform decisions. Australia has labelling standards (FSANZ [18], HSR [19]) and dietary guidelines [12]. However, they do not fully inform consumers about the potential health risks, nor do they provide system-wide, readily translatable information for consumers. Instead, consumers need to translate complex, low-profile, on-pack nutritional information when faced with in-store purchasing decisions. Research has shown that consumers find it difficult to understand and apply such information to their own decisions around food and beverage consumption [20].

The World Health Organization (WHO) has specifically quantified limits for intake of sugars; adults and children should have less than 10% of their daily energy intake from sugar and less than 5% to confer greater health benefit [21]. For the average adult, this translates into 12 teaspoons of sugar as the upper limit, with a limit of 6 teaspoons of sugar better for health. The amount for children varies with age, with upper limits not exceeding the limit for adults (12 teaspoons). One 600 mL (~20 fl. oz.) soda typically contains approximately 16 teaspoons of sugar, thereby exceeding the daily limit for adults. The extent to which consumers are aware of this information is unclear.

Evidence indicates that consumer beliefs about healthiness of different beverages are mixed. It has been shown that consumers consider soda, diet soda and energy drinks to be among the unhealthiest beverage options when choosing among a range of sweetened and unsweetened beverage options [22,23,24,25,26,27]. These perceptions related to concerns about sugar, caffeine and artificial sweetener contents [25,26,27,28,29]. However, results of some studies indicate those who regularly consume SSBs are more likely than infrequent consumers to perceive them as healthy [24,27].

The available literature, all from the US, suggests that there are considerable gaps in people’s knowledge of beverages [24,28,30,31,32,33]. The results may not be generalisable as they are conducted on US samples. Furthermore, the studies are difficult to compare due to the broad range of measures used to assess various facets of beverage knowledge. Inevitably, the different measures of knowledge used across studies has led to mixed findings with respect to levels of knowledge and impact on consumption behaviour. The range of approaches taken to assess knowledge of the nutritional composition of SSBs (e.g., measuring general knowledge such as ‘Is this drink high in sugar?’ compared to specific knowledge such as ‘How many teaspoons of sugar does this drink contain?’) highlights a challenge in establishing the minimum knowledge required for consumers to make informed choices.

A review of the existing literature on SSB knowledge suggests that there are two areas of knowledge that are critical to making informed decisions: understanding the composition of drinks (e.g., energy, sugar) and knowing the potential health effects associated with consumption. The research suggests that people have a better understanding of drinks’ sugar content than energy content [24,28,30,31], but this knowledge does not necessarily predict consumption. Contrasting results have been found, with better knowledge of specific calorie or sugar information not related to consumption in two studies [30,32], related to lower SSB consumption in one study [31] and related to higher soda and diet soda consumption in another [24]. Other studies indicate that health perceptions contribute more to consumption than does knowledge of sugar and calorie content [28,33]. Moreover, a systematic review found that improving knowledge and attitudes related to recommended sugar intake was associated with a reduction in sugar consumption [34].

Regarding potential health effects associated with consumption, qualitative studies conducted across different locations and age groups have shown that there is a belief that weight gain, Type 2 Diabetes, hyperactivity and dental cavities are consequences of SSB consumption [25,35,36]. However, this knowledge is not universal. Estimates of the proportion of people reporting different health effects of SSB consumption from quantitative studies have varied. Several studies conducted in the US indicated that most adult participants identified that SSB consumption, broadly defined, was associated with weight gain (75–84%) [30,37,38,39] and Type 2 Diabetes (71–76%) [37,38,39], whereas far fewer participants identified heart disease (32%) [38,39] and high cholesterol (24–32%) [38,39]. Knowledge of the link between dental caries and SSB consumption has ranged from 57% to 78% across different studies [37,38,39]. Demographic differences in knowledge of health risks associated with SSB consumption have been found, but the pattern of results varies across studies [37,38,39]. Findings of several studies indicate that SSB consumption was not related to knowledge of health risks after controlling for demographic characteristics [37,38,39]. However, SSB consumption was greater among those with a lack of knowledge of the association between SSB consumption and heart disease [38] and those who were ambivalent about the link between SSB consumption and weight gain [30].

Public health interventions aimed at reducing SSB consumption are increasingly targeting consumers’ limited knowledge and perceptions of health risks and excess sugar and calorie intake associated with consumption. Evidence of the effectiveness of strategies such as mass media campaigns and warning labels to curb consumption is increasing [40,41,42] but, to date, such strategies have been difficult to implement due to political and industry barriers [43,44]. Establishing Australian population levels of knowledge regarding SSB and ASB consumption, as well as the degree to which different facets of knowledge are linked to beverage consumption, can inform policy makers of the value and necessity of public health interventions. It can also inform the development of interventions by identifying, for example, which facets of knowledge to target. Further, the establishment of robust population estimates of knowledge can serve as a baseline against which future interventions aimed at increasing understanding and knowledge, such as campaigns and warning labels, can be assessed. To ensure a high level of specificity in assessing participants’ knowledge of the contents of popular and regularly consumed beverages at the population level, and to allow a meaningful comparison between sweetened and artificially sweetened drinks, this study focused on two drink types in particular: soda (regular Coca-cola) and diet soda (artificially-sweetened Coca-cola). The aim of this study was to explore knowledge and beliefs associated with soda and diet soda in the Australian population, providing unprecedented in-depth insight and extending previous work conducted mostly in the US. This study complements another study using the same dataset that reports on the demographic, health, behavioural and environmental correlates of SSBs [45].

## 2. Materials and Methods

This study was conducted on data from a large Australian study on SSB consumption (methods reported in detail in [46]). A national computer-assisted telephone survey of adults aged 18 years and over was conducted between February and April 2017. The sample was obtained via random digit dialling of Australian landline and mobile phones (35:65 split). Calls were made up to 6 (mobile) or 8 (landline) times to achieve an interview. In-scope persons (i.e., aged 18 years and over, able to speak English) were identified using screening questions. In households with multiple eligible persons, the youngest male, followed by the youngest female, was selected to ensure appropriate representation of younger respondents in the sample, who are typically underrepresented in telephone surveys [47]. On average, interview duration was 21 min. Quality control procedures were followed throughout the fieldwork phase, including briefing and monitoring interviewers, and pilot testing the survey to check the flow and clarity of the questions. This study was approved by the University of Adelaide’s Human Research Ethics Committee (Approval code: H-2016-285), and participants provided verbal informed consent prior to commencing the survey.

### 2.1. Measures

Respondents were initially asked to estimate the recommended limit on daily sugar intake for adults and children: How many teaspoons of added sugar do you think a [healthy adult]/[primary school aged child] could have per day to stay within the guidelines? Respondents were then asked a series of knowledge questions about an average 600 mL bottle of soft drink [soda] such as Coke (i.e., Coca-cola) or Sprite. They were asked to numerically estimate: teaspoons of sugar; calories; percentage of daily energy intake (for an average adult); and minutes of jogging it would take to work off the drink (for an average adult), with prompts to encourage estimation if unsure. We estimated correct responses for each question based on the nutritional composition of ‘Coke’. As these were approximate values and there are variations in sugar and calories across drinks, jogging speed, and the rate at which individuals burn off calories, a range of responses was accepted as the correct amount. Specific quantities and ranges were 16 teaspoons of sugar (acceptable range: 11 to 20 teaspoons), 258 calories (acceptable range: 201 to 300), 12% of daily energy (acceptable range: 10 to 14%), and 50 min of jogging (acceptable range: 31 to 60 min), as specified in a previous study [48].

Knowledge of health risks was assessed by asking participants if they knew of any illnesses or health effects associated with drinking (1) soda (like Coke/Sprite) and (2) artificially sweetened (diet) soda (like Coke Zero), with the order of the two drinks randomised. Respondents were asked to list as many illnesses as they could think of and responses were coded into categories (e.g., Type 2 Diabetes, weight gain, tooth decay). Beliefs about the relative healthiness of drinks were assessed by asking respondents ‘Compared to soft drinks [soda] such as Coke, do you think the following drink types are a more healthy choice, less healthy choice or about the same?’ with the following drink types presented in random order: diet soda (like Coke Zero), fruit juice, and sports drinks. General beliefs about health effects of drinking sugary drinks were assessed by asking respondents ‘If an average [adult/child] drank a sugary drink every day, would they be likely or unlikely to have health problems later in life?’ The available responses of very likely, somewhat likely, neither likely nor unlikely, somewhat unlikely, and very unlikely were dichotomised into likely (very or somewhat likely), and did not say likely (neither likely nor unlikely, somewhat unlikely, and very unlikely).

Beverage consumption was assessed by asking respondents how often (presented as number of 250 mL [~8fl. oz.] cups per day, week, month, less than monthly or never) they typically drank each of the following drinks: soda (like Coke/Sprite), sports drinks, energy drinks, fruit juice, and diet soda (like Coke Zero). Interviewers were trained in converting common drink sizes into number of cups in case participants gave answers in other formats (e.g., 1 can is approximately 1.5 cups).

### 2.2. Analysis

Analyses were undertaken with SPSSv26 [49]. Chi-square tests were used to compare knowledge and beliefs according to demographic characteristics (sex, age and education) and beverage consumption profiles based on either frequent (5 or more cups per week), regular (1–4 cups per week), or infrequent (monthly or less) consumption of (1) soda and (2) diet soda. Logistic regression was used to assess the relationship between knowledge and belief variables and consumption while controlling for age, sex and education. There were no differences between frequent and regular consumers in the bi-variate analyses. These consumption categories were combined into ‘regular’, or at least weekly consumption and compared to ‘infrequent’, or monthly or less consumption for ease of analysis and interpretation of the logistic regressions. As is standard for telephone-based population surveys, a two-stage weighting procedure was used. First, a design weight was applied which adjusted for the individual’s chance of selection. Second, a post-stratification weight was applied to ensure the final sample was weighted to relevant population benchmarks for age, gender, location and telephony status (landline vs. mobile). As this study was exploratory, no adjustments were made to p-values for the bivariate analyses. However, conservative cut-off *p*-values were used when interpreting results to account for multiple comparisons.

## 3. Results

In total, 3430 participants were recruited into this study, which represented a participation rate of 44% (i.e., completed interviews as a proportion of in-scope sample members who could be contacted within the call cycle) and a response rate of 16% (i.e., completed interviews as a proportion of all study participants, incomplete participation, refusals, non-contacts and those with unknown eligibility estimated as eligible [50]). The mean age of participants was 46.9 years (SD = 18.3) and 51% were female. A total of 38% held a bachelor degree or higher, which was higher than the national average (26%) [51]. Further demographic characteristics of the sample are available elsewhere [45,46]. Most respondents could report sugar intake guidelines consistent with WHO recommendations: 74% indicated that the adult guideline for teaspoons of sugar was between 0 and 5, 20% indicated that it was between 6 and 12 and 3% indicated it was 13 or more (4% indicated that they did not know). Similar results were obtained in relation to child guidelines: 85% indicated 0–5, 9% indicated 6–12, 1% indicated 13 or more and 4% did not know.

Most participants believed that health problems would develop later in life from daily sugary drink consumption (Table 1). Overall, 71.4% of respondents specified that Type 2 Diabetes was associated with soda consumption whereas the rates for specifying other known health effects were lower: 44.3% named weight gain, 27.7% heart disease and 18.8% tooth decay. The rates of naming health effects associated with diet soda consumption were markedly lower than those for soda consumption (Type 2 Diabetes 30.6%; weight gain 16.4%; heart disease 13.3%; tooth decay 6.2%), except for cancer where the reverse was observed (14.7% for diet sodas and 6.5% for sodas).

As shown in Table 1, most knowledge and belief variables varied significantly according to sex, age and level of education. However, the absolute percent difference and effect sizes (reported as Cramer’s V coefficient ‘V’) were small to medium, so only results with a *p* < 0.001 and only the greatest absolute difference between groups are noted in the following text. A greater proportion of females compared to males reported that soda consumption was associated with Type 2 Diabetes (8% absolute difference; V = 0.09), weight gain (7.8% difference; V = 0.08), heart disease (5.5% difference; V = 0.06), and tooth decay (5.3% difference; V = 0.07). A lower proportion of participants aged 18–30 years compared to those aged 46–60 years reported that soda consumption was associated with Type 2 Diabetes (14.1% absolute difference; V = 0.15), weight gain (17.4% difference; V = 0.13) and tooth decay (9.7% difference; V = 0.09). A lower proportion of participants with high school education or less compared to those with a bachelor degree or higher reported that soda consumption was associated with Type 2 Diabetes (17.1% absolute difference; V = 0.15), weight gain (13.8% difference; V = 0.11), heart disease (13.3%; V = 0.12), tooth decay (8.4%; V = 0.09) and cancer (4.1%; V = 0.07). Reporting that diet soda was associated with cancer occurred at a greater rate for those aged 61 years and over compared to those aged 31–45 years (11.9% difference; V = 0.13) and for the least educated compared to the most educated (9.7% difference; V = 0.14).

Approximately half of participants viewed diet soda (51.9%) and sports drinks (55.3%) as having the same level of healthiness as soda, while 59% indicated that fruit juice was healthier than soda. A greater proportion of males compared to females rated diet soda, sports drinks and fruit juice as healthier than soda, with absolute differences in percentages and effect sizes of 6.4% (V = 0.09), 9.7% (V = 0.12) and 8.4% (V = 0.10), respectively. The most educated participants had a higher rate of reporting diet sodas as healthier than sodas than the least educated participants (5.2% absolute difference; V = 0.06). Compared to the most educated participants, the least educated participants had a higher rate of reporting sports drinks as less healthy (11.4% absolute difference) and a lower rate of reporting sports drinks as the same as soda (12.4%; V = 0.09). The youngest participants were more likely than the oldest participants to rate sports drinks as healthier than sodas (15.3% absolute difference; V = 0.13).

Overall, few participants were able to correctly specify nutrition-related information for a 600 mL bottle of soda. Specifically, 31.1% of respondents estimated the correct number of teaspoons of sugar (approx. 11–20 teaspoons), whereas 56.5% reported fewer teaspoons of sugar than the correct amount (1–10 teaspoons). Underestimation occurred more often among those aged 18–30 compared to those aged 31–45 (12.8% absolute difference; V = 0.12) and the least compared to most educated participants (5.7% difference; V = 0.08). Only 11.0% of participants could correctly specify total calories (approx. 201–300 calories), whereas overestimates (301 calories and over; 34.1%) or ‘don’t know’ responses (38.4%) were more common. Similarly, only 9.3% of participants could correctly specify percent daily calories (10–14%) whereas 58.9% reported a larger percent than the correct amount. Reporting the correct amount of total calorie information occurred at a higher rate among the youngest compared to oldest participants (8.9% absolute difference; V = 0.15) and those with higher versus lower levels of education (5.4% difference; V = 0.11). In terms of estimates of the number of minutes of jogging that would be required to work off the energy consumed from a 600 mL beverage, 37.3% of participants specified the correct amount of time (approx. 31 to 60 min) and 32.3% reported 30 min or less (i.e., underestimation). Underestimating jogging time occurred at a higher rate among males compared to females (9% absolute difference; V = 0.10), those aged 61 and over compared to those aged 46–60 years (10.2% difference; V = 0.12) and those with lower versus higher levels of education (6.3% difference; V = 0.09).

Knowledge and beliefs were associated with soda consumption. Results of bivariate analyses comparing frequent (5 cups or more per week), regular (1–4 cups per week) and infrequent (monthly or less) soda consumers are displayed in Supplementary Table S1. Compared to frequent and/or regular soda consumers, infrequent SSB consumers were significantly more likely to agree that sugary drink consumption increased the likelihood of health problems for adults (12.5% absolute difference; V = 0.09) and for children (7.4% difference; V = 0.07) and to report a range of health effects associated with consumption, including Type 2 Diabetes (8.9% difference; V = 0.7), weight gain (12.1% difference; V = 0.09; tooth decay (9.0% difference; V = 0.07) and cancer (3.8% difference; V = 0.07). Infrequent SSB consumers also had a significantly higher rate of indicating that sports drinks had the same level of healthiness as sodas (9.4% difference; V = 0.06). Multi-variate results comparing regular to infrequent soda consumers are reported in Table 2, and results with a p-value below 0.01 are noted below. After controlling for sex, age and education, regular soda consumers had greater odds of not reporting cancer (OR = 2.18; 95%CI = 1.48–3.21) as a health effect associated with soda consumption. They also indicated that sports drinks were less healthy (OR = 1.34; 95%C = 1.08–1.66) than sodas; and, in reference to a 600 mL bottle of soda, substantially overestimated total calories (OR = 1.76; 95%CI = 1.28–2.43), and did not know the number of minutes of jogging required to work off the calories consumed (OR = 1.85; 95%CI = 1.19–2.89).

Knowledge and beliefs were also associated with diet soda consumption (see Table 3). After controlling for sex, age and education, regular diet soda consumers, compared to infrequent diet soda consumers, were significantly less likely report Type 2 Diabetes as a health effect associated with diet soda consumption (OR = 1.48; 95% Confidence Interval (CI) = 1.16–1.88). Regular diet soda consumers were also 3.7 (95% CI = 2.99–4.53) times as likely as infrequent diet soda consumers to indicate that diet sodas were healthier than sodas. Bi-variate results are presented in Supplementary Table S2.

## 4. Discussion

The aim of this study was to establish baseline levels of knowledge and beliefs regarding the health effects associated with soda and diet soda consumption in the Australian population. The results showed that while most Australians had a general appreciation that daily consumption of sugary drinks would likely lead to future health problems, more detailed knowledge of potential health effects was largely lacking, which may be because this type of information is not readily accessible to consumers. However, most participants were unable to accurately report or even approximate the correct amounts of sugar or energy contained in a popular drink choice (i.e., a 600 mL bottle of Coke), which is currently displayed on the packaging via a nutrition panel and energy symbols. This suggests that consumers do not pay adequate attention to this type of information, which limits their ability to apply it to their own context, such as the amount of exercise required to ‘work it off’. These large gaps in community knowledge about products that are consumed by Australians in substantial quantities have important implications for consumer rights to information as well as for public health.

It is important to note that the high levels of agreement with the general proposition that consumption among adults (80%) or children (90%) could lead to health problems observed in this study were not matched by a high level of understanding about what those health effects might be. In terms of specific health effects, Type 2 Diabetes was widely reported (by 71%), but weight gain (44%), heart disease (28%) and even tooth decay (19%) were not reported by the majority. Knowledge of these potential health effects was significantly lower among younger and less-educated groups; these groups are also more likely to be regular consumers [2].

Our findings are consistent with other studies. A focus group discussion among Australian university students showed that Type 2 Diabetes featured most prominently among numerous health risks discussed as consequences of soda consumption [35]. Other studies conducted in the USA [37,38,39,52] have shown similar results for Type 2 Diabetes, but knowledge of weight gain and dental caries was much higher than observed in the current study, which is likely due to prompted assessment methods used as opposed to the unprompted measure employed in the current study where respondents were simply asked to report any health effects. Assessing knowledge in this way (unprompted) is a better measure of saliency and top-of-mind knowledge that may be more likely to influence decisions about purchase and consumption [40,53]. Nevertheless, results of some studies have indicated that the rates of agreement (prompted) for conditions such as high cholesterol, heart disease and hypertension were much lower than for conditions such as Type 2 Diabetes, weight gain and dental caries [38,39]. Overall, these findings indicate that interventions aimed at increasing awareness of the multiple, specific risks of health conditions are warranted.

The findings suggest that interpretable information, which is not currently on packaging but is promoted through other channels, such as teaspoons of sugar or exercise equivalent metrics, was retained by more people than the abstract information that is currently on packaging such as number of calories or percent of daily caloric intake information. Qualitative research has shown that people have difficulty understanding and interpreting energy information [36], despite having ready access to this information via on-pack nutrition information panels. Furthermore, experimental research has shown that concrete visual images of sugar are better understood than abstract information [54]. Large proportions of respondents in this study underestimated, rather than overestimated the sugar content and exercise equivalent metric, which has important implications for consumers’ decision-making processes. Inaccurate or minimal understanding of the nutritional composition of sugary drinks may inadvertently put people at risk of overconsumption and may lead to decision-making that has negative implications for overall food intake and attempting to maintain a healthy weight [55]. Furthermore, it is unlikely that consumers will be able to make informed decisions regarding limiting their sugar intake to stay within health recommendations when the information provided is in abstract units (e.g., grams), rather than more tangible units such as teaspoons.

This study appears to be the first to explore knowledge of health effects associated with sodas compared to diet sodas. The results indicated that most participants (79%) did not perceive diet sodas as a healthier alternative to regular (sugar-sweetened) soda. This is consistent with findings of other studies indicating that diet sodas and non-diet sodas are both rated as unhealthy [22,24,27,56]. This study provides additional insight as to why diet sodas are perceived as unhealthy. Respondents generally reported that the health conditions associated with sodas were also associated with diet sodas, albeit to a lesser extent. However, an exception was cancer, which was associated with diet sodas at a higher rate (14.7%) than regular soda (6.5%). Cancer is among the most feared health conditions [57], and reports on the links between artificial sweeteners and cancer are commonly reported in the media [58]. Given that added sugar is also a growing concern among the general public, consumers may be increasingly confused about what is safe to consume [23,25,26,27,59]. Nonetheless, the results of this study indicate regular consumers of diet sodas are more likely than infrequent consumers to perceive them to be healthier than regular sodas. This is an important public health matter given that the population is expected to weigh up the risks of their dietary actions and act accordingly. However, emergent evidence indicates limited health benefit and possible health harm associated with switching from a sugary drink to a diet version [13,14].

The implications of encouraging consumers to switch drinks are further reflected in the results of this study in the comparison of relative healthiness of other sugary drinks. Sports drinks were considered equivalent to sodas, whereas fruit juice was considered as healthier. People may be inclined to increase consumption of other beverages when they are encouraged to move away from soda, and they are likely to move to beverages that they consider to be healthier. Participants may choose other sugary drinks or ASBs as alternatives to soda and may not necessarily switch to the optimal alternative beverage, water. There may be a need for interventions that aim to reduce consumption of soda and/or sugary drinks to also inform or reiterate the optimal beverage choice(s) to consumers.

There is clearly a gap between nutrition guidance and consumers’ health knowledge and beliefs. Calorie[kilojoule]/energy content information is made available on beverage packages in Australia by law, but it is clear that people do not access and absorb this information in its current format. Conversely, there is evidence that people are aware of adverse health effects, but these are not necessarily front-of-mind when making decisions about what to consume. Changing the format of labels on sugary drink bottles is one important option for increasing people’s knowledge about the risks of consuming sugary drinks at the point of making consumption decisions. Various experiments have been conducted to test whether knowledge of health effects or sugar content increases after exposure to this information, and whether this additional knowledge influences intentions to purchase a sugary drink in a hypothetical setting [60,61,62,63,64]. Results of these studies show the potential of warning labels in improving understanding of health harms associated with consumption of SSBs and reducing the likelihood of purchasing SSBs. More research is needed on the most appropriate information to present to consumers that will influence them to reduce their consumption.

Another type of intervention that can be effective at increasing knowledge is mass media public education campaigns. Barragan and colleagues showed that campaign exposure increased the likelihood of reporting the correct quantity of sugar in a soda [41]. Other studies have shown that exposure to health effects messages increased agreement that too much sugar causes health problems [53] and intentions to reduce sugary drink consumption [53,65,66]. Some studies have also found subgroup differences in responses to campaigns; Robles [65] found that ‘moderate’ rather than ‘heavy’ soda consumers were more likely to reduce soda intake after campaign exposure, and Morley et al. [40] found that knowledge of health effects increased for overweight SSB consumers after campaign exposure, but the effect was not observed for the population as a whole. It is likely that the type of message is also important, with fear appeals more likely to induce behaviour change than humorous or nurturing advertisements [40,67]. An intervention aimed at increasing levels of health literacy related to SSBs has also been trialled that showed positive impact on attitudes, perceptions and intentions towards reducing SSB intake [68]. Price and tax interventions are also likely to play a role in reducing consumption [61], and multi-faceted approaches are likely to be ultimately required to achieve real behaviour change [43].

The current study has strengths and limitations. This exploratory study provides a detailed examination of soda and diet soda knowledge and beliefs by adapting measures used in previous studies that have good face validity [30,31,52,53,69]. However, further validation would be beneficial and would strengthen the results of this study. Expanding this study to include other types of drinks would also be beneficial to allow comparisons across drink categories. Data were collected on a range of other potentially relevant co-variates that were not central to and beyond the scope of this study, but may be worthy of further exploration in future studies. Examples include socio-economic status, Body Mass Index, physical activity, health literacy, and understanding of other sources of information about drinks. All responses were self-report and are at risk of response biases. However, interviews were anonymous to reduce the influence of social desirability bias. Furthermore, it was a cross-sectional survey limited to one time point for capturing responses, which may be influenced by temporary external influences, such as articles in the media and advertising campaigns about the harms of excess sugar consumption. Analyses were not adjusted for multiple comparisons as this study was exploratory, and while conservative *p*-values were used to interpret the results, additional dedicated studies are needed to confirm the results.

## 5. Conclusions

Overall, this study showed that most respondents had some awareness of the types of health risks associated with soda consumption and believed that long-term consumption would cause problems later in life. Respondents also perceived health risks from diet sodas, but these views were less prevalent. Most respondents were unable to accurately describe the sugar and energy content of an average bottle of soda. While a substantial proportion of respondents demonstrated awareness regarding limits for sugar consumption as per guidelines (0 and 5 teaspoons per day), they appeared to be substantially less aware of how this relates to the amount of sugar in an average bottle of soda, putting them at risk of overconsumption of sugar and increased chance of experiencing health effects later in life. The Australian population would benefit from public health interventions, such as social marketing campaigns and on-product health warning labels, that increase understanding of the sugar content and the health risks that are associated with soda consumption, and the potential health risks associated with diet soda consumption.

## Figures and Tables

**Table 1:** Soda-related knowledge and beliefs by sex, age and education (*n* = 3430).

	Overall	Sex			Age (Years)					Education			
		Male	Female	χ^2^ *p*-Value; Cramer’s V	18–30	31–45	46–60	61+	χ^2^ *p*-Value; Cramer’s V	High School or Less	Vocation or Partial University	Bachelor Degree or Higher	χ^2^ *p*-Value; Cramer’s V
	% ±95% Confidence Interval	%	%		%	%	%	%		%	%	%	
Overall		49.2	50.8		23.8	23.9	25.6	25.8		27.3	33.6	38.0	
**Likelihood of Future Health Problems from Daily Soda Consumption**													
Likely for adults	79.9 ± 3.0	**75.0**	**84.9**		**85.6**	**83.5**	78.4	**73.3**		78.4	79.2	82.2	
Not likely for adults	19.9 ± 1.5	**25.0**	**15.1**	<0.001; 0.12	**14.4**	**16.5**	21.6	**26.7**	<0.001; 0.12	21.6	20.8	17.8	0.054; 0.04
Likely for children	89.9 ± 3.2	**86.3**	**93.4**		**93.3**	90.1	88.8	**87.9**		90.1	89.2	90.7	
Not likely for children	10.1 ± 1.1	**13.7**	**6.6**	<0.001; 0.12	**6.7**	9.9	11.2	**12.1**	0.002; 0.07	9.9	10.8	9.3	0.442; 0.02
**Illnesses or Health Effects Reported as Being Associated with Consumption (Response Options Were Not Provided) ^a^**													
Soda													
Type 2 Diabetes	71.4 ± 2.8	67.4	75.4	<0.001; 0.09	65.3	76.8	79.4	64.6	<0.001; 0.15	62.1	70.8	79.2	<0.001; 0.15
Weight gain	44.3 ± 2.2	40.3	48.1	<0.001; 0.08	34.7	48.0	52.1	41.9	<0.001; 0.13	37	42.9	50.8	<0.001; 0.11
Heart disease	27.7 ± 1.8	24.9	30.4	<0.001; 0.06	31.2	33.1	27.9	18.9	<0.001; 0.12	20.1	27.3	33.4	<0.001; 0.12
Tooth decay	18.8 ± 1.5	16.1	21.4	<0.001; 0.07	14.6	19.4	24.3	16.7	<0.001; 0.09	14.2	18.5	22.6	<0.001; 0.09
Hypertension	8.2 ± 1.0	8.3	8.0	0.795; 0.00	7.9	10.3	8.9	5.9	0.009; 0.06	5.8	8.7	9.5	0.005; 0.06
Cancer	6.5 ± 0.9	5.1	7.9	0.001; 0.06	6.4	9.3	6.4	4.3	0.001; 0.07	4.5	6	8.6	<0.001; 0.07
Unsure	11.0 ± 1.1	13.4	8.7	<0.001; 0.08	14.7	7.5	6.1	15.5	<0.001; 0.13	17.2	10.8	6.4	<0.001; 0.14
Diet Soda													
Type 2 Diabetes	30.6 ± 1.9	28.1	33.0	0.002; 0.05	30.4	28.2	30.8	33	0.202; 0.04	31.6	30.2	30.4	0.765; 0.01
Weight gain	16.4 ± 1.4	15.0	17.7	0.032; 0.04	13.6	15.0	17.9	18.8	0.013; 0.06	15.3	16	17.3	0.433; 0.02
Heart disease	13.3 ± 1.2	11.5	15.0	0.003; 0.05	16.7	14.4	11.4	10.8	0.001; 0.07	10.2	13.8	14.9	0.005; 0.06
Tooth decay	6.2 ± 0.8	5.7	6.8	0.184; 0.02	5.5	6.3	6.9	6.3	0.694; 0.02	5.8	5.6	7.2	0.184; 0.03
Hypertension	4.4 ± 0.7	3.6	5.2	0.017; 0.04	4.3	5.9	4.3	3.5	0.126; 0.04	3.2	4.9	5	0.093; 0.04
Cancer	14.7 ± 1.3	12.8	16.5	0.002; 0.05	13.5	19.9	17.2	8	<0.001; 0.13	9.1	14.6	18.8	<0.001; 0.11
Unsure	38.0 ± 2.1	42.3	33.9	<0.001; 0.09	40.1	37.5	34.0	41	0.013; 0.06	45.5	37.4	33.2	<0.001; 0.10
**Relative Healthiness of Drinks: Compared to Soda**													
Diet sodas are more healthy	21.5 ± 1.6	**25.2**	**18.8**	<0.001; 0.09	20.0	22.7	23.0	22.6	0.004; 0.05	20.3	**19.4**	**25.5**	<0.001; 0.06
less healthy	25.3 ± 1.7	26.6	25.0		25.8	25.4	**21.8**	**29.9**		**28.8**	**28**	**21.7**	
the same	51.3 ± 2.4	**48.2**	**56.3**		54.1	51.9	**55.2**	**47.5**		50.9	52.6	52.7	
Fruit juices are more healthy	58.5 ± 2.6	**63.4**	**55.0**	<0.001; 0.10	61.6	56.3	**55.1**	**63.1**	0.002; 0.06	58.4	**55.7**	**62.5**	0.004; 0.05
less healthy	5.4 ± 0.8	6.2	4.8		5.0	5.3	5.3	6.2		6.3	6.5	**4.2**	
the same	35.0 ± 2.0	**30.4**	**40.2**		33.3	38.4	**39.6**	**30.6**		35.3	**37.8**	**33.3**	
Sports drinks are more healthy	21.7 ± 1.6	**27.1**	**17.4**	<0.001; 0.12	**31.3**	23.1	**18.8**	**16**	<0.001; 0.13	23.1	21.5	22.1	<0.001; 0.09
less healthy	20.7 ± 1.5	20.5	22.0		**16.7**	**15.3**	21.2	**31.3**		**27.3**	22.2	**15.9**	
the same	55.3 ± 2.5	**52.4**	**60.7**		**52.0**	**61.6**	**60.0**	**52.7**		**49.6**	56.4	**62**	
**In Reference to a 600 mL Bottle of Soda**													
Teaspoons of Sugar				0.282; 0.03					<0.001; 0.12				<0.001; 0.08
Approx. correct (11 to 20)	31.1 ±1.9	30.1	32.1		**26.3**	**38.5**	**34.9**	**25.4**		**28.0**	30.6	**33.8**	
Underestimate (1 to 10)	56.5 ± 2.5	58.1	54.9		**62.6**	**49.8**	**50.0**	**63.1**		**60.6**	55.1	54.9	
Overestimate (21+)	9.9 ± 1.1	9.5	10.2		10.3	11.1	**13.0**	**5.4**		**7.2**	**12.0**	10.1	
Don’t know	2.5 ± 0.5	2.3	2.8		**0.7**	**0.6**	2.2	**6.1**		**4.3**	2.3	**1.2**	
Total Calories				0.015; 0.06					<0.001; 0.15				<0.001; 0.11
Approx. correct (201 to 300)	11.0 ± 1.1	10.1	11.8		**14.5**	11.4	12.6	**5.6**		**8.8**	**9.5**	**14.2**	
Underestimate (0 to 200)	16.5 ± 1.4	16.9	16.0		**22.2**	16.0	**12.4**	15.9		17.2	**14.6**	17.9	
Overestimate (301 to 600)	17.3 ± 1.4	16.6	18.1		**20.8**	**21.0**	18.3	**10.3**		**13.4**	16.6	**21.1**	
Large over estimate (601+)	16.8 ± 1.4	**18.8**	**14.8**		18.6	**19.5**	17.4	**11.8**		**14.2**	**19.0**	16.8	
Don’t know	38.4 ± 2.1	37.6	39.2		**24.0**	**32.1**	39.3	**56.4**		**46.4**	40.2	**30.0**	
Percent Daily Calories				<0.001; 0.09					<0.001; 0.18				<0.001; 0.15
Approx. correct (10 to 14)	9.3 ± 1.0	**10.5**	**8.1**		**14.6**	9.4	**6.2**	**7.3**		9.4	8.7	10.0	
Underestimate (0 to 9)	7.4 ± 0.9	7.8	7.0		6.4	6.6	7.6	8.9		7.9	7.1	7.2	
Overestimate (15 to 40)	29.2 ± 1.8	**32.3**	**26.5**		**39.5**	**34.7**	28.5	**16.1**		**22.6**	**27.1**	**36.6**	
Large overestimate (41+)	29.7 ± 1.8	**27.9**	**31.6**		**26.3**	**33.8**	**33.6**	**25.4**		**24.0**	**33.7**	30.5	
Don’t know	24.2 ± 1.6	**21.6**	**26.8**		**13.2**	**15.5**	24.1	**42.4**		**36.0**	23.4	**15.7**	
Minutes of Jogging to Work Off				<0.001; 0.10					<0.001; 0.12				<0.001; 0.09
Approx. correct (31 to 60)	37.3 ± 2.0	**34.9**	**39.8**		39.5	**40.7**	37.9	**31.9**		34.8	37.2	**39.6**	
Underestimate (1 to 30)	32.3 ± 1.9	**36.9**	**27.9**		33.9	29.7	**27.9**	**38.1**		**35.1**	34.5	**28.8**	
Overestimate (61 to 90)	5.6 ± 0.8	5.6	5.6		5.9	6.4	5.8	**4.0**		4.7	4.9	**6.6**	
Large overestimate (91+)	19.6 ± 1.5	**17.8**	**21.4**		19.5	20.2	**24.3**	**15.1**		**16.9**	19.2	**22.0**	
Don’t know	5.0 ± 0.7	4.8	5.3		**1.2**	**3.1**	4.1	**11.0**		**8.4**	4.2	**3.0**	

Note: *p*-values are the result of chi-square tests. Bold cells are statistically significant (based on adjusted standardised residuals). ^a^ Very small proportions of participants recalled depression as associated with soda (0.7%; *n* = 23) and diet soda (0.3%; *n* = 9) consumption. Approx. = approximately

**Table 2:** Logistic regression predicting soda consumption based on soda-related knowledge and beliefs.

	Logistic Regression: Regular Soda Consumption (1) vs. Infrequent Soda Consumption (0)					
	Unadj Odds Ratio	95% Confidence Interval	*p*-Value	Adj Odds Ratio ^†^	95% Confidence Interval	*p*-Value
	*n* = 3275			*n* = 3225		
**Likelihood of Health Problems from Soda Consumption**						
Not likely for adults	1.14	(0.92–1.42)	0.235	1.23	(0.97–1.56)	0.081
Likely for adults (Ref)	1			1		
Not likely for children	1.14	(0.86–1.52)	0.368	1.13	(0.83–1.53)	0.443
Likely for children (Ref)	1			1		
**Illnesses or Health Effects Associated with Soda Consumption**						
Type 2 Diabetes not reported	1.34	(1.12–1.59)	0.001	1.14	(0.95–1.38)	0.164
Reported (Ref)	1			1		
Weight gain not reported	1.37	(1.17–1.61)	<0.001	1.19	(1.00–1.41)	0.045
Reported (Ref)	1			1		
Heart disease not reported	1.11	(0.93–1.33)	0.256	1.17	(0.96–1.42)	0.115
Reported (Ref)	1			1		
Tooth decay not reported	1.36	(1.11–1.68)	0.004	1.19	(0.96–1.49)	0.115
Reported (Ref)	1			1		
Hypertension not reported	0.82	(0.62–1.08)	0.151	0.79	(0.59–1.06)	0.112
Reported (Ref)	1			1		
Cancer not reported	2.09	(1.44–3.04)	<0.001	2.18	(1.48–3.21)	<0.001
Reported (Ref)	1			1		
**Relative Healthiness of Drinks: Compared to Soda**						
Fruit juices are more healthy	1.20	(1.01–1.42)	0.039	1.21	(1.01–1.46)	0.036
less healthy	1.34	(0.95–1.90)	0.099	1.30	(0.90–1.88)	0.169
the same (Ref)	1			1		
Sports drinks are…more healthy	1.42	(1.17–1.72)	<0.001	1.14	(0.93–1.39)	0.217
less healthy	1.16	(0.95–1.42)	0.141	1.34	(1.08–1.66)	0.008
the same (Ref)	1			1		
**In Reference to a 600 mL Bottle of Soda**						
Teaspoons of Sugar						
Underestimate (1 to 10)	1.18	(1.98–1.40)	0.074	1.22	(1.01–1.47)	0.043
Overestimate (21+)	1.08	(0.81–1.44)	0.591	0.99	(0.73–1.33)	0.926
Don’t know	0.87	(0.48–1.58)	0.646	1.04	(0.53–2.03)	0.902
Approx. correct (11 to 20) (Ref)	1			1		
Total Calories						
Underestimate (0 to 200)	1.29	(0.95–1.75)	0.104	1.36	(0.98–1.87)	0.065
Overestimate (301 to 600)	1.21	(0.89–1.64)	0.221	1.22	(0.88–1.68)	0.226
Large overestimate (601+)	1.75	(1.29–2.37)	<0.001	1.76	(1.28–2.43)	0.001
Don’t know	1.18	(0.89–1.56)	0.255	1.35	(1.00–1.82)	0.053
Approx. correct (201 to 300) (Ref)	1			1		
Percent Daily Calories						
Underestimate (0 to 9)	0.80	(0.54–1.17)	0.250	1.06	(0.70–1.59)	0.791
Overestimate (15 to 40)	1.31	(0.98–1.75)	0.064	1.35	(1.00–1.83)	0.051
Large overestimate (41+)	0.99	(0.74–1.33)	0.950	1.21	(0.88–1.65)	0.236
Don’t know	0.84	(0.61–1.15)	0.277	1.23	(0.88–1.72)	0.236
Approx. correct (10 to 14) (Ref)	1			1		
Minutes of Jogging to Work Off						
Underestimate (1 to 30)	1.12	(0.93–1.35)	0.238	1.11	(0.91–1.35)	0.316
Overestimate (61 to 90)	0.81	(0.56–1.16)	0.250	0.80	(0.54–1.19)	0.273
Large overestimate (91+)	1.05	(0.84–1.30)	0.688	1.08	(0.86–1.36)	0.518
Don’t know	1.46	(0.96–2.22)	0.075	1.85	(1.19–2.89)	0.007
Approx. correct (31 to 60) (Ref)	1			1		

^†^ Adjusted for sex, age and education; Ref = reference group, Approx. = approximately.

**Table 3:** Logistic regression predicting diet soda consumption based on diet soda-related knowledge and beliefs.

	Logistic Regression: Regular Diet Soda Consumption (1) vs. Infrequent Diet Soda Consumption (0)					
	Unadj Odds Ratio	95% Confidence Interval	*p*-Value	Adj Odds Ratio ^†^	95% Confidence Interval	*p*-Value
	*n* = 3353			*n* = 3297		
**Illnesses or Health Effects Associated with Diet Soda Consumption**						
Type 2 Diabetes not reported	1.50	1.18–1.91	0.001	1.48	1.16–1.88	0.001
Reported (Ref)	1			1		
Weight gain not reported	1.39	1.04–1.88	0.029	1.35	1.00–1.82	0.050
Reported (Ref)	1			1		
Heart disease not reported	1.12	0.81–1.55	0.487	1.11	0.80–1.54	0.521
Reported (Ref)	1			1		
Tooth decay not reported	0.88	0.59–1.32	0.537	0.89	0.60–1.33	0.574
Reported (Ref)	1			1		
Hypertension not reported	0.73	0.45–1.18	0.205	0.75	0.47–1.21	0.242
Reported (Ref)	1			1		
Cancer not reported	0.75	0.59–0.96	0.023	0.77	0.60–0.99	0.044
Reported (Ref)	1			1		
**Relative Healthiness of Drinks: Compared to Soda**						
Diet sodas are more healthy	3.68	3.00–4.52	<0.001	3.68	2.99–4.53	<0.001
less healthy	0.72	0.56–0.93	0.012	0.73	0.56–0.94	0.016
the same	1			1		

^†^ Adjusted for sex, age and education; Ref = reference group.

## Supplementary Materials

The following are available online at https://www.mdpi.com/2072-6643/12/9/2841/s1, Table S1: Knowledge and beliefs related to soda consumption (*n* = 3430), Table S2: Knowledge and beliefs related to diet soda consumption.
