# Supplementary material for: An In-Depth Exploration of Knowledge and Beliefs Associated with Soda and Diet Soda Consumption
Source: Nutrients. 2020 Sep 17;12(9):2841. doi: 10.3390/nu12092841 (PMC7551593; doi:10.3390/nu12092841)
Supplement: Supplementary file 1 [file nutrients-12-02841-s001.pdf]

Table S1. Knowledge and beliefs related to soda consumption (*n*=3430).

	Overall	Soda consumption			χ^2 p-value	Cramer' s V
		**Frequent (5 or more cups per week)**	**Regular (1 to 4 cups per week)**	**Infrequent (Monthly or less)**		
	%	%	%	%		
Overall		8.8	20.2	70.9		
**Likelihood of health problems from sugary drink consumption**						
Likely for adults	79.9	**68.8**	80.8	**81.3**		
Not likely for adults	19.9	**31.2**	19.2	**18.7**	<0.001	0.09
Likely for children	89.9	**83.4**	89.9	**90.8**		
Not likely for children	10.1	**16.6**	10.1	**9.2**	<0.001	0.07
**Illnesses or health effects associated with soda consumption**						
Diabetes …reported	71.4	**64.5**	**67.7**	**73.4**		
…not reported	28.5	**35.5**	**32.3**	**26.6**	<0.001	0.07
Weight gain …reported	44.3	**34.9**	**39.2**	**47.0**		
…not reported	55.7	**65.1**	**60.8**	**53.0**	<0.001	0.09
Heart disease …reported	27.7	23.7	25.4	28.9		
…not reported	72.3	76.3	74.6	71.1	0.051	0.04
Tooth decay …reported	18.8	**11.0**	17.5	**20.0**		
…not reported	81.2	**89.0**	82.5	**80.0**	<0.001	0.07
Hypertension …reported	8.2	9.0	8.7	7.9		
…not reported	91.8	91.0	91.3	92.1	0.721	0.01
Cancer …reported	6.5	4.3	**3.8**	**7.6**		
…not reported	93.5	95.7	**96.2**	**92.4**	<0.001	0.07
**Relative healthiness of drinks: compared to soda**						
Fruit juices are…more healthy	58.5	58.7	**64.4**	**57.7**		
…less healthy	5.4	7.7	5.9	5.0		
…the same	35.0	33.6	**29.7**	**37.3**	0.002	0.05
Sports drinks are…more healthy	21.7	25.3	**27.9**	**20.2**		
…less healthy	20.7	24.9	21.4	20.6		
…the same	55.3	49.8	**50.7**	**59.2**	<0.001	0.06
**In reference to a 600ml bottle of soda…**						
**Teaspoons of sugar**						
Approx correct (11 to 20)	31.1	27.1	28.5	32.3		
Underestimate (1 to 10)	56.5	60.9	60.5	54.9		
Overestimate (21+)	9.9	9.4	9.2	10.0		
Don't know	2.5	2.7	1.7	2.7	0.091	0.04
**Total calories**						
Approx correct (201 to 300)	11.0	**6.4**	10.7	11.7		
Underestimate (0 to 200)	16.5	13.7	18.8	16.1		
Overestimate (301 to 600)	17.3	16.7	16.8	17.6		
Large over estimate (601+)	16.8	19.7	**20.5**	**15.3**		
Don't know	38.4	43.5	**33.2**	39.3	<0.001	0.07
**Percent daily calories**						
Approx correct (10 to 14)	9.3	11.0	8.9	9.2		
Underestimate (0 to 9)	7.4	9.0	5.7	7.7		
Overestimate (15 to 40)	29.2	30.6	**35.1**	**27.5**		
Large overestimate (41+)	29.7	27.9	27.9	30.6		
Don't know	24.2	21.6	22.5	25.0	0.009	0.05
**Minutes of jogging to work off**						
Approx correct (31 to 60)	37.3	34.1	37.1	37.9		
Underestimate (1 to 30)	32.3	**39.1**	32.8	31.3		
Overestimate (61 to 90)	5.6	2.7	5.6	5.9		
Large overestimate (91+)	19.6	**16.7**	19.9	20.0		
Don't know	5.0	7.4	4.5	4.9	0.039	0.05

Bold cells are statistically significant (based on adjusted standardised residuals).

.

Table S2. Knowledge and beliefs related to diet soda consumption.

		Diet soda consumption				
	**Overall**	**Frequent (5 or more cups per week)**	Regular (1 to 4 cups per week)	Infrequent (Monthly or less)	χ^2 p-value	Cramer′ s V
	%	%	%	%		
Overall		6.6	11.5	81.9		
**Illnesses or health effects associated with DIET soda consumption**						
Diabetes …reported	30.6	**15.4**	**23.2**	**32.9**		
…not reported	69.4	**84.6**	**76.8**	**67.1**	<0.001	0.11
Weight gain …reported	16.4	**7.1**	**12.7**	**17.7**		
…not reported	83.6	**92.9**	**87.3**	**82.3**	<0.001	0.08
Heart disease …reported	13.3	**4.4**	12.0	**14.2**		
…not reported	86.7	**95.6**	88.0	**85.8**	<0.001	0.07
Tooth decay …reported	6.2	4.0	6.6	6.4		
…not reported	93.7	96.0	93.4	93.6	0.329	0.03
Hypertension …reported	4.4	2.2	4.8	4.5		
…not reported	95.6	97.8	95.2	95.5	0.237	0.03
Cancer …reported	14.7	15.0	**19.1**	**14.1**		
…not reported	85.3	85.0	**80.9**	**85.9**	0.033	0.05
**Relative healthiness of drinks: compared to soda…**						
Diet sodas are…more healthy	21.5	**57.1**	**39.6**	**16.6**		
…less healthy	25.3	**8.8**	**18.5**	**28.2**		
…the same	51.3	**34.1**	**41.9**	**55.3**	<0.001	0.21

Bold cells are statistically significant (based on adjusted standardised residuals).
